# A Triple-Band Reflective Polarization Conversion Metasurface with High Polarization Conversion Ratio for Ism and X-Band Applications

**DOI:** 10.3390/s22218213

**Published:** 2022-10-26

**Authors:** Badisa Anil Babu, Boddapati Taraka Phani Madhav, Sudipta Das, Niamat Hussain, Syed Samser Ali, Nam Kim

**Affiliations:** 1 Department of Electronics and Communication Engineering, GMR Institute of Technology, Razam 532127, India; 2 Antennas & Liquid Crystals Research Centre, Koneru Lakshmaiah Education Foundation, Vaddeswaram 522302, India; 3 Department of Electronics & Communication Engineering, IMPS College of Engineering and Technology, Malda 732103, India; 4 Department of Smart Device Engineering, Sejong University, Seoul 05006, Korea; 5 Department of Electronics & Communication Engineering, University Institute of Technology, Burdwan 713104, India; 6 Department of Information and Communication Engineering, Chungbuk National University, Cheongju 28644, Korea

**Keywords:** industrial scientific and medical (ISM), X-band, polarization conversion ratio (PCR), reflective metasurface (RM), triple-band

## Abstract

A compact and triple-band polarization converting reflective type metasurface (PCRM) with a high polarization conversion ratio (PCR) is proposed for strategic wireless antenna-integrated applications. The unit cell of the metasurface is composed of S- and G-shaped patches separated with a parasitic gap and the grounded via is connected to the full ground plane. The unit cell is etched on an FR4 substrate (dielectric constant, ε_r_ = 4.4, loss tangent, tan δ = 0.02), with compact dimensions of 10 mm^3^ × 10 mm^3^ × 1.6 mm^3^. This structure provides a resonance at 5.2 (ISM), 6.9, and 8.05 GHz (X-band) frequencies. The designed unit cell structure is studied for Transverse Electric (TE)/Transverse Magnetic (TM) incident waves and their responses to the various incident angles. The corresponding PCR is calculated, which shows 92% in the lower frequency band (5.2 GHz), 93% in the second frequency band (6.9 GHz), and 94% in the high-frequency band (8.05 GHz). The total efficiency of the structure shows 83.2%, 62.95%, and 64.6% at the respective resonance bands. A prototype of the proposed PCRM with 3 × 3 unit cells is fabricated to validate the simulated results. The experimental data agrees with the simulation results. The compactness, triple-band operation with a high PCR value of more than 92% makes use of the designed metasurface in wireless antenna-integrated applications at ISM and X-bands.

## 1. Introduction

Metasurfaces (MS) are artificially engineered two-dimensional structures, which have the capability of manipulating Electromagnetic (EM) propagation behavior. The MS consists of sub-wavelength periodic structures called unit cells [1,2]. The controllable characteristic parameters of EM waves are amplitude and phase. Thus, MS can be utilized for the realization of functionalities like polarization, gain control, and amplitude control [3]. The applications of these functionalities are used to realize perfect absorbers [4], frequency selective surfaces (FSS) [5], AMC reflectors and polarization converters [6] based on epsilon negative, and epsilon near to zero properties [7]. The metasurface is highly efficient in acquiring low-profile, high-efficiency, and broadband polarization converters [8]. Polarization converters have distinct properties in controlling polarization and they have been utilized in the microwave [9], terahertz [10], and even optical [11] range of frequencies.

Polarization conversion is one of the functionalities required for current wireless devices to control polarization, gain, radar cross-section (RCS), etc. The realization of polarization converting MS can be done in two operating modes: transmission [12] and reflection [13]. Based on the type of polarization conversion, each mode is categorized into linear to circular, circular to linear, linear to linear, and cross-polarization conversion MS [14,15,16]. Most of the realized polarization-converting MSs are limited to broadband [17], ultra-wideband [18], wideband [19], and dual-band operation only [20]. However, to meet the current demand for wireless devices, there is a need to extend functionality to multiband. Various multifunctional and multi-polarization conversion metasurfaces were realized in the literature using different reconfiguration mechanisms [21,22,23]. These metasurfaces provided a challenge in terms of active component integration and the loss generated by these active components. Hence the synthesis of compact, passive polarization-converting metasurfaces have become the solution to the above challenge. Only a few works have reported on multi-band passive polarization converting MS with compact size, high polarization conversion ratio (PCR) values, and high performance [24,25,26,27,28,29,30].

In this paper, the design of a triple-band cross-polarization converting metasurface based on reflection is illustrated with compact dimensions and high PCR values. The unit cell structure resonates at 5.2 GHz, 6.9 GHz, and 8.05 GHz frequencies. The corresponding polarization conversion ratio is 92%, 93%, and 94%, respectively. The total efficiencies are 83.2%, 62.95%, and 64.6% at respective operating bands. For the incidence and polarization angles of the incident wave, the structure provides angular stability up to 30° incident angles, and it acts as a polarization-sensitive metasurface for polarization angles. Similarly, the characteristics of TM and TE incidents on the surface are studied. The simulated characteristics are experimentally validated using a 3 × 3 PCRM fabricated prototype. The compactness, high PCR, and triple-band feature make it suitable for the implementation in strategic antenna-integrated applications for ISM and X-bands.

## 2. Design and Simulation Analysis

### 2.1. Unit Cell Configuration

**Figure 1:**
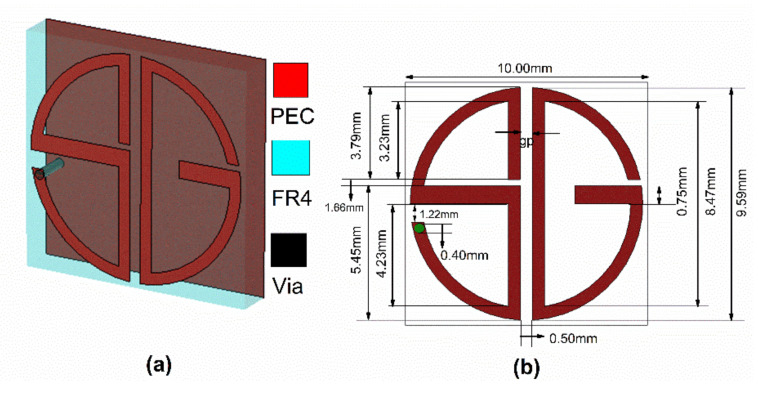
(**a**) Perspective view of the Unit cell; (**b**) Unit cell dimensions.

A schematic diagram of the reflective type cross-polarization converting metasurface with the simulated setup is shown in Figure 1a. A single-layer FR4 lossy is used as a substrate with dielectric constant (ε_r_) = 4.4 and loss tangent (tan δ) = 0.002. S-and G-shaped metal patches separated with a parasitic gap and grounded via to the S-shape that makes the structure resonate at 5.2, 6.9, and 8.05 GHz. The unit cell dimensions are 10 mm^3^ × 10 mm^3^ × 1.6 mm^3^. The geometric dimensions of the unit cell are shown in Figure 1b. The numerical simulation software CST MWS is used for design and simulated analysis.

The simulation setup consists of a linearly polarized plane wave incidence in the Z-direction, while the unit cell structure lies in an XY plane. The reflected electric fields consist of co-polarization (S_11_ & S_22_) and cross-polarization components (S_12_ & S_21_), which are related using Equation (1):

(1) $$\left[\begin{array}{l} E_{x}^{r}\\ E_{y}^{r} \end{array}\right]=\left(\begin{array}{cc} r_{xx} & r_{yy}\\ r_{yx} & r_{yy} \end{array}\right)\left[\begin{array}{l} E_{x}^{i}\\ E_{y}^{i} \end{array}\right]$$

*i*—incident wave; *r*—reflected wave; where $r_{xx}=\left|\frac{E_{x}^{r}}{E_{x}^{i}}\right|$ and $r_{yx}=\left|\frac{E_{y}^{r}}{E_{x}^{i}}\right|$.

**Figure 2:**
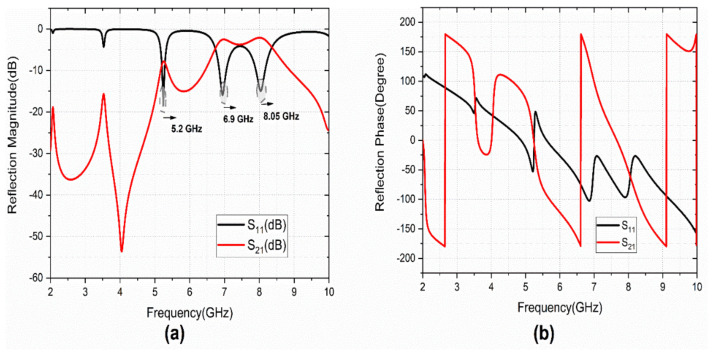
Simulated unit cell Co-and Cross-polarized Reflection characteristics (**a**) magnitude, (**b**) phase.

The investigation of the cross-polarization conversion behavior of the proposed structure is carried out under linear polarized incidence. Figure 2a,b depicts the simulated reflection characteristics of the design. The structure provides three co-polarized (S_11_) resonating bands at 5.2, 6.9, and 8.05 GHz.

**Figure 3:**
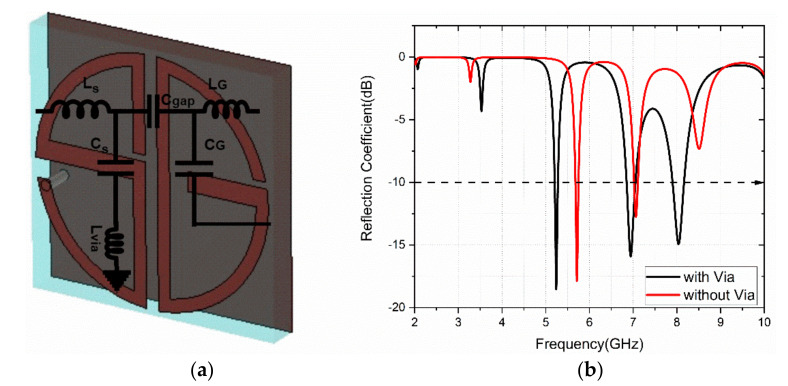
(**a**) Unit cell equivalent LC-circuit model representation; (**b**) Comparison of S_11_ with and without via.

The cross-polarized reflection coefficient (S_21_) also has peaks at operating bands. The reflection phase of the co-and cross-polarized component gives the reflected phase details for the linearly polarized incident wave. The phase difference between the co-and cross-polarized components indicates that the cross-polarization conversion mechanism of the designed metasurface to operate in triple-bands. The simulated analysis is carried out with CST MWS software. The equivalent inductance (L) and capacitance (C) values for the S-shape patch are L_s_ and C_s_. Similarly, L_G_ and C_G_ are the equivalent values for G-shape. The L_via_ is the inductance of grounded via. Then, the resonance of the structure element is calculated as: $f_{r}=\frac{1}{2\pi \sqrt{\mathrm{LC}}}$.The equivalent LC-circuit model for the design is presented in Figure 3a. The role of via in the design is studied with the simulated S_11_ (dB), as shown in Figure 3b. It is observed that the resonant frequency is lowered with triple-band operation.

**Figure 4:**
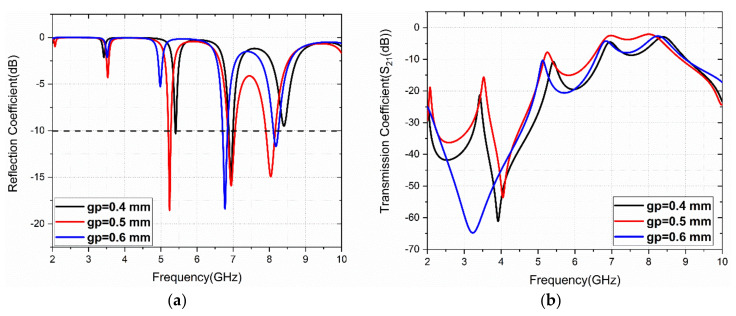
Parametric analysis of gap (gp) between S-and G-patches: (**a**) S_11_ (dB), (**b**) S_21_ (dB).

The physical significance of the structure is studied using a parametric analysis of the gap (gp variable) between the S-and G-shaped patches. The simulated S_11_ and S_21_ results for gap variation are presented in Figure 4. It is observed that the optimized gap is obtained at gp = 0.5 mm for triple-band operation.

### 2.2. Response to the Incident Angle and Polarization Angle Variation

**Figure 5:**
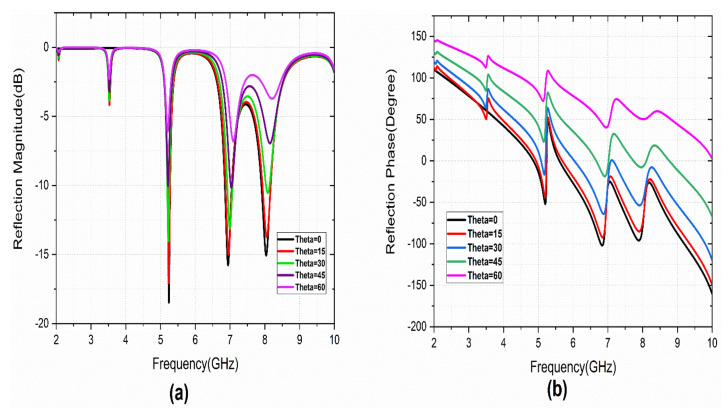
S_11_ variation with Theta variation: (**a**) magnitude, (**b**) phase.

**Figure 6:**
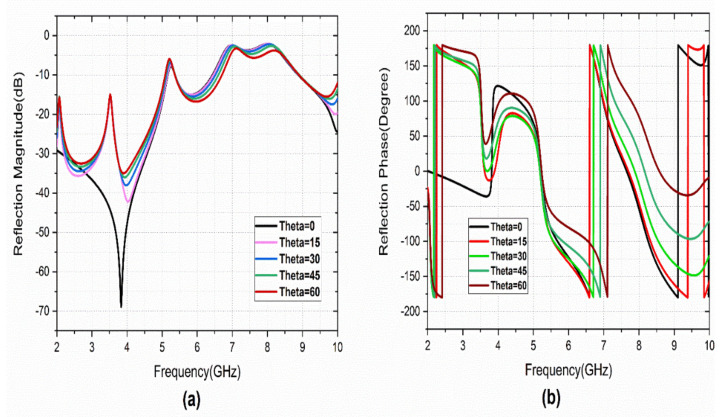
S_21_ variation with Theta variation: (**a**) magnitude, (**b**) phase.

The reflection characteristics of the metasurface under different incident angles (theta) and polarization angles are studied. The simulated theta and phi variations are performed with a variation from 0° to 90° a step width of 15°. Figure 5 illustrates the co-polarized reflection characteristics. It provides angular stability of up to 30° theta variations. Figure 6 represents the reflection characteristics of the cross-polarization reflection coefficient. A few minor variations are observed with theta variation.

**Figure 7:**
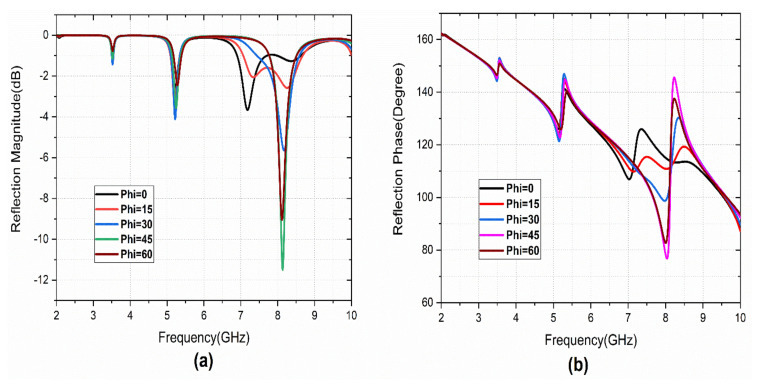
Reflection characteristics under Phi variation: (**a**) magnitude, (**b**) phase.

The metasurface is studied further for polarization angle variation. The structure is polarization-sensitive, as the reflection characteristics are not matched with the normal incident wave. The evidence for the simulation results is shown in Figure 7. The simulation results show that the metasurface is polarization-sensitive and provides the cross-polarization conversion operation with the angular stability of 30° of incident angle.

### 2.3. Analysis with TE and TM Incident Wave

**Figure 8:**
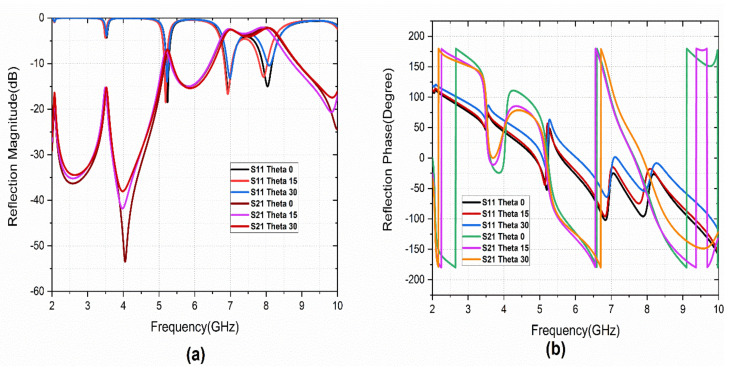
Reflection characteristics under TE incident angle variation: (**a**) magnitude, (**b**) phase.

The reflection characteristics of the PCRM with vertically polarized (TE) and horizontally polarized (TM) wave incident angle (theta) variation is studied. Figure 8 illustrates the reflection characteristics of the metasurface under TE incidence. It is observed that small variations in reflection magnitude and phase are observed. For these variations, a corresponding variation in S_21_ is also observed, which also gives the PCRM the ability to work as a polarization converter under TE incident angle variation.

**Figure 9:**
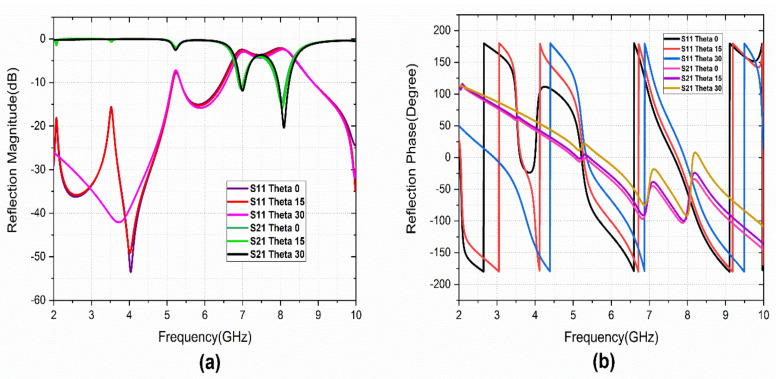
Reflection characteristics under TM incident angle variation: (**a**) magnitude (**b**) phase.

Under the TM incident wave, the metasurface is affected by the fundamental mode of resonance because this resonance is provided by the magnetic resonance of the structure. For the other higher resonant frequencies, similar results are observed as in TE incident angle variations. Figure 9 shows the reflection characteristics under TM theta variation.

### 2.4. Analysis of Surface Current Distribution

**Figure 10:**
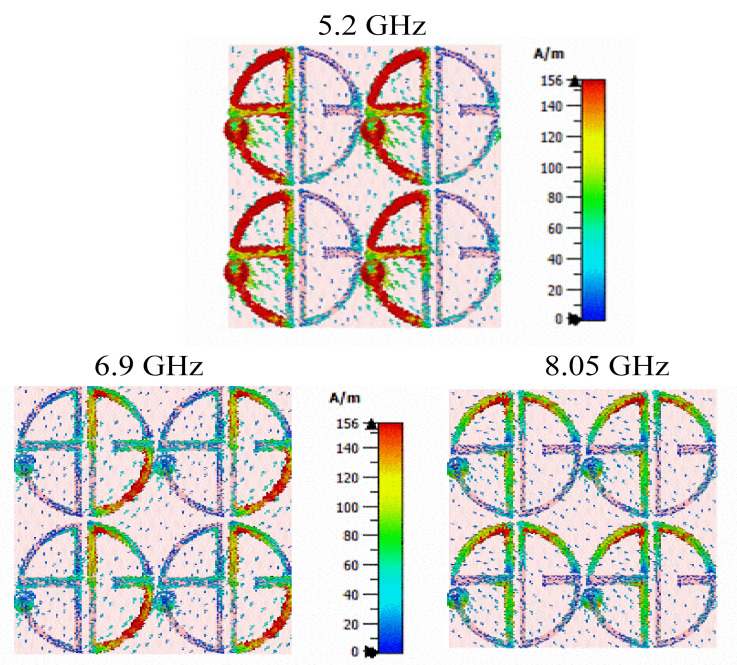
Simulated Surface current distribution at 5.2, 6.9, and 8.05 GHz.

The resonance phenomenon and the cross-polarization conversion mechanism can be studied using surface current distribution. Figure 10 illustrates the surface current distribution at the three resonant frequencies. The surface current is mostly distributed on the S-shape with the via and patch current direction being out of phase with the ground, which causes magnetic resonance at 5.2 GHz. The current intensity is on the G-shape with an in-phase current direction at 6.9 GHz, which makes electric resonance. Whereas the higher-order resonance is generated by both the S-and G-shape patches. Thus, the lower polarization conversion band is provided by the S-shape, and the second mode is due to the G-shape and the higher mode is generated by both the S-and G-shape with the parasitic gap.

#### Performance Analysis

The cross-polarization conversion efficiency of the design is quantified in terms of PCR. PCR can be calculated from the co-and cross-polarized reflection magnitudes as represented in Equation (2):

(2) $$\mathrm{PCR}=\frac{r_{yx}^{2}}{r_{yx}^{2}+r_{xx}^{2}}=\frac{r_{xy}^{2}}{r_{xy}^{2}+r_{yy}^{2}}$$

**Figure 11:**
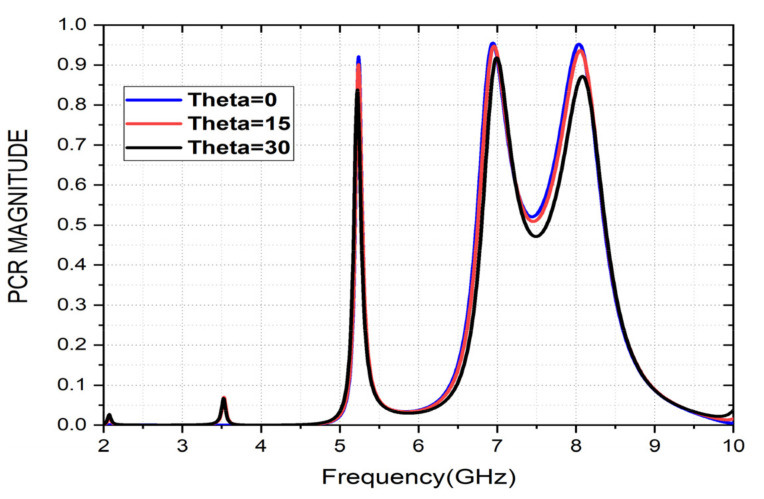
PCR Variation for various incident angles (theta).

*r_yx_* represents a cross-polarized component, i.e., S_21_, and *r_xx_* represents the co-polarization component, i.e., S11. The highest polarization conversions obtained are 92% for the first operating band, 94% for the second operating band, and 93% for the third operating band under normal incidence. The PCR variation with oblique angles of the incident wave (theta) is also calculated and presented in Figure 11. Incident angles of up to 30° provides a PCR value of more than 85%.

## 3. Experimental Setup and Results

**Figure 12:**
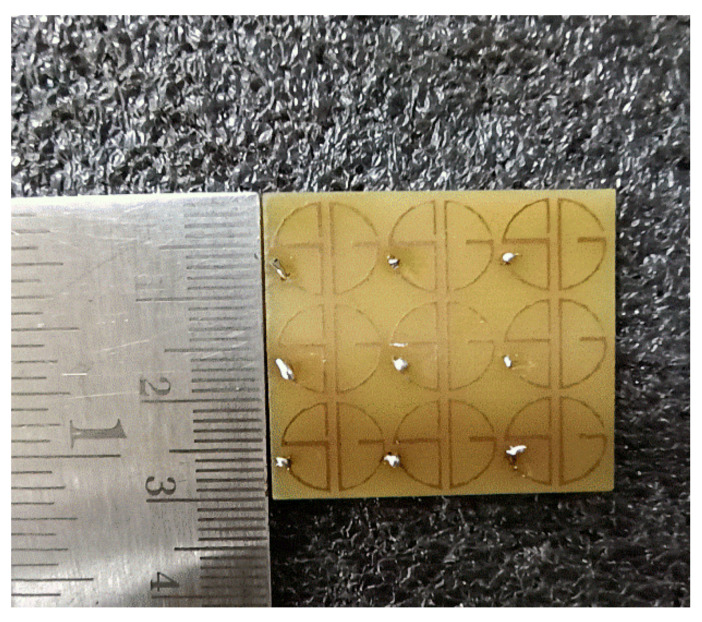
Fabricated 3 × 3-unit cell prototype.

**Figure 13:**
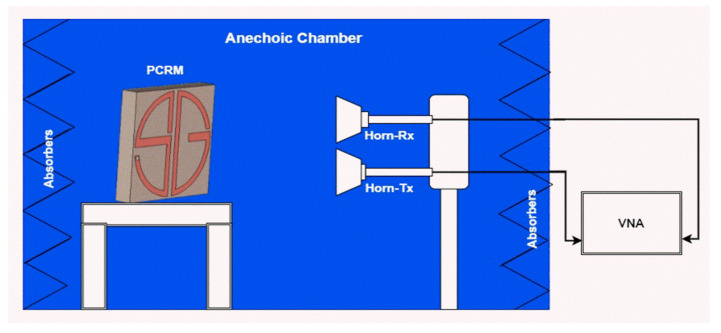
Schematic of the measurement setup.

**Figure 14:**
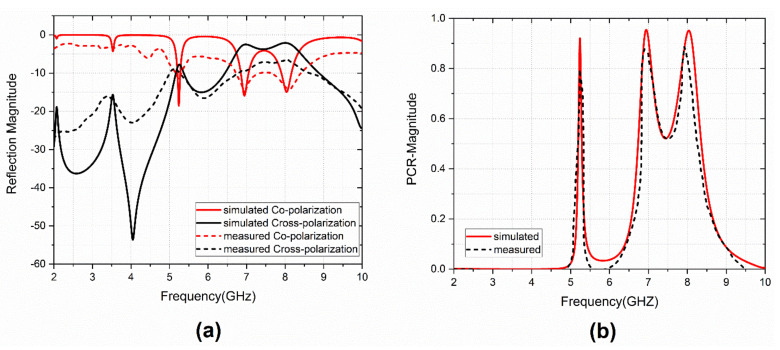
Comparison between simulated and measured results: (**a**) reflection magnitude, (**b**) PCR.

To validate the design, a 3 × 3 experimental sample was fabricated and measured, and the co-and cross-polarization reflection characteristics were calculated, as well as the PCR value. The fabricated sample is given in Figure 12. The schematic diagram of the experimental setup for characterization is shown in Figure 13. Anritsu MS2037C VNA and two standard horn antennas were used as transmitters and receivers with x-and y-polarization. The measured and calculated results agreed well. The observed variations in measured results were due to fabrication and measurement errors only. Figure 14 shows the comparison between simulated and measured results of the reflection magnitude and PCR under the normal incidence of the incoming wave. The variation in measured results was due to fabrication errors and size limitations of the prototype in the measurement setup. The measured results were taken at normal incidence angles.

**Figure 15:**
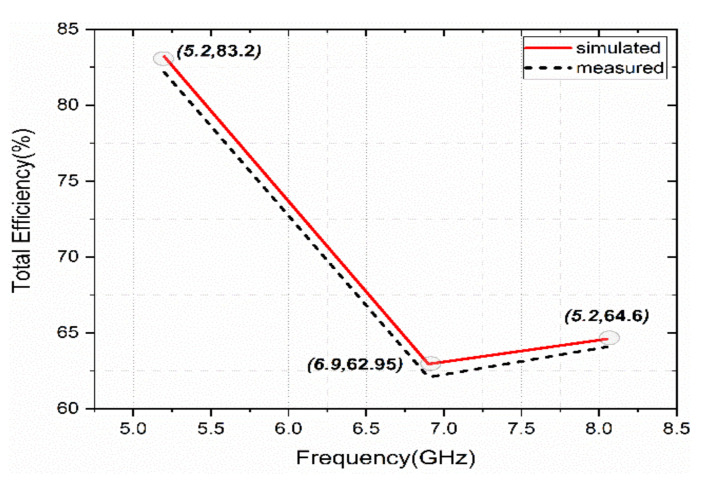
Total efficiency as a function of frequency.

The simulated and measured total efficiency is shown in Figure 15. The measured data shows similar efficiency curves as the numerically computed total efficiency. However, the measured efficiency was observed to be a little lower than the simulated values due to substrate losses.

Table 1 presents a comparison of the designed triple-band reflective cross-polarization converter with the state-of-the-art related works reported in the literature. It can be observed that the proposed metasurface provides a triple-band operation with a relatively compact size. The structure consists of parasitic patch elements with grounded via that acted as a cross-polarization converter with PCR of >92%. Moreover, it offers high PCR values, which is one of the essential requirements for the current wireless communication systems. The physical mechanism of the realized structure is verified using numerical simulations and experimental characterization of a 3 × 3 array sample. There are few works that have a smaller size [24,29]. However, ref. [24] did not perform an analysis of PCR, while ref. [29] has lower PCR values.

## 4. Conclusions

In this article, a triple-band, compact PCRM is designed, and its characteristics are studied under incidence and polarization angles. The physical significance of the structural elements is presented along with an equivalent LC-circuit model representation and a detailed parametric analysis. The simulated results show that it provides angular stability of up to 300 of incidence angle and is sensitive to the polarization angle. It provides PCR values of 92%, 93%, and 94% at 5.2, 6.9, and 8.05 GHz. The PCR values under TE and TM modes are also calculated, and the operating mechanism is demonstrated through surface current distribution. The total efficiency of reflecting PCRM is 83.2%, 62.95%, and 64.6%, at resonating bands. These results are compared with the existing triple-band reflecting metasurface in terms of angular stability, unit cell size, and PCR. The features, such as compactness and triple-band operation with PCR values, have strategic antenna-integrated applications for ISM and X-band communications.

## Figures and Tables

**Table 1:** Comparison with other related works.

Ref. No	Substrate	Unit Cell Size (L × W × h) λ_0_^3^	PCR (%)	Frequency (GHz)	Conversion Type
[24]	FR4	0.128 × 0.128 × 0.128	_	9.1–16.5, 20–25.4, 17.4–18.9	Cross-polarization LP-CP
[25]	FR4	0.19 × 0.19 × 0.049	>86	4.47–5.35 9.45–13.60	Cross-polarization LP-CP LP-LP
[26]	F4B	0.285 × 0.285 × 0.071	>90	10.75, 19.65, 27.35	Cross-polarization LP-LP
[27]	RT Duroid 4730	0.186 × 0.186 × 0.055	≥80	4.34–4.98, 6.77–6.97, 8.25–8.69, 10.72–15.56	Cross-polarization LP-CP
[28]	F4B-2	0.28 × 0.28 × 0.084	≥90	4.60–12.27, 13.79–13.90, 14.61–14.69, 15.44–15.52	Cross-polarization LP-LP
[29]	F4B	0.152 × 0.152 × 0.038	>90	5.7,8.8,14.85	Multi polarization CP-LP, LP-CP LP-LP
[30]	Rogers RO 40003	0.5 × 60.56 × 0.034	>90	5.30–5.41, 5.77–7.58, 9.27–13.91, 17.53–19.59	Multifunctional LP-LP LP-CP
Prop. Work	FR4	0.173 × 0.173 × 0.027	>92	5.2, 6.9, 8.05	Cross-Polarization LP-LP

λ_0_ is the center fundamental frequency wavelength, LP—Linear Polarization, CP—Circular Polarization.

## Data Availability

Not applicable.
